# Supplementary material for: Estimating the impact of drug use on US mortality, 1999-2016
Source: PLoS One. 2020 Jan 15;15(1):e0226732. doi: 10.1371/journal.pone.0226732 (PMC6961845; doi:10.1371/journal.pone.0226732)
Supplement: S1 Appendix — (DOCX) [file pone.0226732.s001.docx]

S1 Appendix. Modeling the association between drug-coded mortality and mortality from all other causes of death

For this model, the outcome variable is the number of deaths from all causes other than drugs (i.e., all ICD-10 codes except F11-F16, F18, F19, X40-X44, X60-64, X85, and Y10-Y14) for a given state-year-age group-sex divided by the number of person-years of exposure (i.e., the death rate, $M_{-D}$). We use negative binomial regression to model the expected value of the logged death rate $\left( \ln M_{-D} \right)\boldsymbol{,}$fit separately by sex:

$\text{ln} \boldsymbol{M}_{\boldsymbol{-}\mathbf{D}}\boldsymbol{=}\boldsymbol{\beta}_{\mathbf{a}}\boldsymbol{X}_{\mathbf{a}}\boldsymbol{+}\boldsymbol{\beta}_{\mathbf{s}}\boldsymbol{X}_{\mathbf{s}}\boldsymbol{+}\boldsymbol{\beta}_{\mathbf{t}}\boldsymbol{T+}\boldsymbol{\beta}_{\mathbf{D}}\boldsymbol{M}_{\mathbf{D}}\boldsymbol{+}\boldsymbol{\beta}_{\mathbf{Da}}\boldsymbol{(}\boldsymbol{M}_{\mathbf{D}}\boldsymbol{\times}\boldsymbol{X}_{\mathbf{a2}}\boldsymbol{)}$ . (1)

The model includes a set of control variables designed to capture background mortality: $\boldsymbol{X}_{\mathbf{a}}$ represents a set of dummy variables for each age group to account for the age pattern of mortality (which is assumed to be fixed over time and across states); $\boldsymbol{X}_{\mathbf{s}}$ is a set of dummy variables for each state to capture across-state differences in overall mortality levels (which are assumed to be fixed over time); and $T$ is a linear term for calendar year (centered at 1999) to account for the underlying trend in mortality. The key predictor of interest is the drug-coded (i.e., ICD-10 codes F11-F16, F18, F19, X40-X44, X60-64, X85, and Y10-Y14) mortality rate ($M_{D}$), which is interacted with age (i.e., we include the product of $M_{D}$and age) to allow the relationship between drug-coded mortality and mortality from other causes to vary across age. For the purposes of multiplying $M_{D}$ by age, we aggregate ages 15-24 as the reference group and collapse ages 65 and older (i.e., we constrain our estimated drug coefficient to be constant within the youngest and oldest age groups). Thus, in Eq. 1, $\boldsymbol{X}_{\mathbf{a}\boldsymbol{2}}$ is a set of dummy variables for ages 25-29, 30-34,...60-64, and 65+. This model assumes a log-linear relationship between $M_{-D}$ and its predictors (i.e., a unit increase in $M_{D}$ is associated with a constant proportional increase in $M_{-D}$).
