# Supplementary material for: Estimating the impact of drug use on US mortality, 1999-2016
Source: PLoS One. 2020 Jan 15;15(1):e0226732. doi: 10.1371/journal.pone.0226732 (PMC6961845; doi:10.1371/journal.pone.0226732)
Supplement: S2 Appendix — (DOCX) [file pone.0226732.s002.docx]

# S2 Appendix. Modeling the association between drug-coded mortality, smoking mortality, and mortality from all other causes of death

In this alternative model, we add lung cancer (i.e., ICD-10 codes C33 and C34) mortality (as a proxy for the impact of smoking) to the model. The outcome is changed to the mortality rate from all causes other than lung cancer or drugs (i.e., all ICD-10 codes except C33-C34, F11-F16, F18, F19, X40-X44, X60-64, X85, and Y10-Y14) $(M_{-\mathrm{DL}}\boldsymbol{)}$ for a given state-year-age group-sex. The model takes the form:

$\mathbf{ln} \boldsymbol{M}_{\boldsymbol{-}\mathbf{DL}}\boldsymbol{=}\boldsymbol{\beta}_{\mathbf{a}}\mathbf{X}_{\mathbf{a}}\boldsymbol{+}\boldsymbol{\beta}_{\mathbf{s}}\boldsymbol{X}_{\mathbf{s}}\boldsymbol{+}\boldsymbol{\beta}_{\mathbf{t}}\mathbf{T}\boldsymbol{+}\boldsymbol{\beta}_{\mathbf{D}}\boldsymbol{M}_{\mathbf{D}}\boldsymbol{+}\boldsymbol{\beta}_{\mathbf{Da}}\left( \boldsymbol{M}_{\mathbf{D}}\boldsymbol{\times}\boldsymbol{X}_{\mathbf{a2}} \right)$

$\boldsymbol{+}\boldsymbol{\beta}_{\mathbf{L}}\boldsymbol{M}_{\mathbf{L}}\boldsymbol{+}\boldsymbol{\beta}_{\mathbf{La}}\left( \boldsymbol{M}_{\mathbf{L}}\boldsymbol{\times}\boldsymbol{X}_{\mathbf{a3}} \right)$ , (2)

where $M_{L}$ is the death rate from lung cancer and $\left( M_{L}\boldsymbol{\times}\boldsymbol{X}_{\mathbf{a}\boldsymbol{3}} \right)$ represents $M_{L}$ multiplied by a set of age dummies$.$ Lung cancer death rates are very low below age 50 and highest at ages 70 and older. Consequently, for the product of $M_{L}$ and age, we aggregate ages 15-49 as the reference group (i.e., we constrain the lung cancer coefficient to be constant within ages 15-49) and collapse ages 80 and older because, although lung cancer mortality is likely to increase with age, we do not expect the relationship between lung cancer and other types of mortality to change appreciably above age 80. Thus, $\boldsymbol{X}_{\mathbf{a3}}$ is a set of dummy variables for ages 50-54, 55-59,...80+.
