# Supplementary material for: Estimating the impact of drug use on US mortality, 1999-2016
Source: PLoS One. 2020 Jan 15;15(1):e0226732. doi: 10.1371/journal.pone.0226732 (PMC6961845; doi:10.1371/journal.pone.0226732)
Supplement: S3 Appendix — (DOCX) [file pone.0226732.s003.docx]

# S3 Appendix. Estimating the drug-associated fraction

The procedure described in the main text for estimating the fraction of other deaths associated with drug use is equivalent to implementing the following formula:

$\boldsymbol{A}_{\boldsymbol{-}\mathbf{D}}\boldsymbol{=1-}\boldsymbol{e}^{\boldsymbol{-}\boldsymbol{\beta}_{\mathbf{D}}^{\mathbf{'}}\left( \boldsymbol{M}_{\mathbf{D}} \right)}$ , (3)

where $\beta_{D}^{'}=\beta_{D}+\beta_{\mathrm{Da}}$(our estimated values of $\beta_{D}^{'}$ are given in Table 1). Thus, the coefficient in this expression, $\beta_{D}^{'}$, includes the main coefficient of $M_{D}$ in Eq. 2 as well as the interactions between $M_{D}$ and age. When $\beta_{D}^{'}$ is positive, $A_{-D}$ lies between 0 and 1. Yet, at some of the oldest ages, $\beta_{D}^{'}$ is negative (Table 1), which suggests a substitution effect (i.e., higher levels of drug-coded mortality are associated with lower levels of “other” drug-associated mortality). Such a substitution effect could result from interstate variation in reporting (e.g., medical examiners/physicians in some states may be more reluctant than in those in other states to ascribe a death to drugs). Since drug-coded mortality is highest in midlife, we focus most of our analysis on the age range 15-64, where $\beta_{D}^{'}$ is positive (in the model that includes both drugs and smoking).^[[1]](#footnote-1)^

Finally, the overall drug-associated fraction for deaths from all causes is a weighted average:

$\boldsymbol{A}_{\mathbf{D}}\boldsymbol{=}\frac{\boldsymbol{D}_{\mathbf{D}}\boldsymbol{+}\boldsymbol{A}_{\boldsymbol{-}\mathbf{D}}\boldsymbol{D}_{\boldsymbol{-}\mathbf{DL}}}{\boldsymbol{D}}$ (4)

where $D_{D}$, $D_{-\mathrm{DL}}$*,* and $D$ represent the observed number of drug-coded deaths, number of deaths from other causes of death except those coded to drug use or lung cancer, and deaths from all causes combined, respectively.

###

1. In cases where $\beta_{D}^{'}$ was negative in the model that includes both drugs and smoking, the value of $A_{-D}$ (representing the reduction in other deaths as a result of drug use) was between -0.02 and zero in 98.7% of cases. The only cases where $A_{-D}$ was less than -0.05 occurred in Alaska among women aged 85+ in 1999; men aged 85+ in 2003 & 2012; and men aged 80-84 in 2008. The lowest value of $A_{-D}$ was -0.086 among Alaskan men aged 85+ in 2012. [↑](#footnote-ref-1)
