# Supplementary material for: Estimating the impact of drug use on US mortality, 1999-2016
Source: PLoS One. 2020 Jan 15;15(1):e0226732. doi: 10.1371/journal.pone.0226732 (PMC6961845; doi:10.1371/journal.pone.0226732)
Supplement: S4 Appendix — (DOCX) [file pone.0226732.s004.docx]

# S4 Appendix. Estimating life expectancy at age 15 in the absence of drug use

For each state and for the US as a whole, we apply standard life table methods to the observed mortality rates by sex, five-year age group (with an open age interval for ages 85 and older), and year to compute sex-specific period life tables. From the resulting period life tables, we then extract the estimates of life expectancy at age 15 ($e_{15}$**)** and the probability of dying between age 15 and 65 ($q[15,65])$ for each year, sex, and geographic area (i.e., state or US as a whole). These estimates represent the observed values based on recorded mortality conditions.

We then re-estimate “adjusted” versions of these same quantities in the absence of drug use by multiplying the observed mortality rates by $1-A_{D}$, where $A_{D}$ represents the estimated fraction of all-cause deaths (in the given sex, age, year, and state/US) associated with drug use based on the model that includes smoking (Eq. 2). Next, we recompute the period life tables to obtain adjusted values of $e_{15}^{-D}$ and $q^{-D}[15,65]$ in the absence of drug use. To estimate the loss in life expectancy at age 15 associated with drug use, we subtract the observed $e_{15}$ from the adjusted $e_{15}^{-D}$. To estimate the proportion of Americans dying between age 15 and 65 as a result of drug use, we subtract the adjusted $q^{-D}\left[ 15,65 \right]$ from the observed $q\left[ 15,65 \right].$

When computing $e_{15}^{-D}$, we allow drug use to influence all death rates even above age 65 where $A_{D}$ may be negative. When $A_{D}$is positive (which is true for all ages between 15 and 64), then the “adjusted” mortality rates in the absence of drug use will be lower than the observed rates, thus increasing life expectancy. Yet, in some cases, $A_{D}$ is negative above age 65. The number of drug-associated deaths resulting from other causes ($A_{-D}D_{-\mathrm{DL}})$ is negative above age 65 because $\beta_{D}^{'}$ and, by extension, $A_{-D}$ are negative. That negative effect is offset, at least in part, by drug-coded deaths ($D_{D})$, which are always positive. Nonetheless, if $-\left( A_{-D}D_{-\mathrm{DL}} \right)>D_{D}$, then $A_{D}$ will be negative (see Eq. 4). In such cases, the “adjusted” rates will be *higher* than the observed rates, which means the contribution to life expectancy will be negative (i.e., eliminating drug use would appear to improve survival at those older ages).
