# Supplementary material for: Estimating the impact of drug use on US mortality, 1999-2016
Source: PLoS One. 2020 Jan 15;15(1):e0226732. doi: 10.1371/journal.pone.0226732 (PMC6961845; doi:10.1371/journal.pone.0226732)
Supplement: S5 Appendix — (DOCX) [file pone.0226732.s005.docx]

# S5 Appendix. Cause-specific models

Drug-coded mortality accounted for 0.8% of all deaths at ages 15+ in 1999, but grew to 2.4% in 2016. Among deaths at ages 15+, lung cancer accounted for 6.5% in 1999, but fell to 5.5% in 2016. We classified the remaining causes of death into the following groups (all percentages refer to deaths at ages 15+):

1. Mental/behavioral disorders, excluding drug-related (ICD-10 codes F00-F10, F17, F20-F99), which comprised 1.7% of deaths in 1999, 4.8% in 2016);
2. Ill-defined causes (R00-R99; 1.0% of deaths in 1999, 1.1% in 2016);
3. Infectious and parasitic diseases (A00-B99; 2.5% of deaths in 1999, 2.5% in 2016);
4. Respiratory diseases (J00-J47, J60-J98, U04, G45; 10% of deaths in 1999 & 2016);
5. Digestive diseases (K00-K93; 3.5% of deaths in 1999, 3.9% in 2016);
6. External causes, excluding drug-related (V01-X39, X45-X59, X65-X84, X86-Y09, Y15-Y98; 5.4% of deaths in 1999, 6.1% in 2016);
7. Circulatory diseases (I00-I99; 41% of deaths in 1999, 31% in 2016);
8. Neoplasms, excluding lung cancer (C00-C32, C37-C97, D00-D48; 17% of deaths in 1999 & 2016);
9. Endocrine, nutritional, & metabolic diseases (E00-E90; 3.9% of deaths in 1999 & 2016);
10. All other causes (7.0% of deaths in 1999, 11.3% in 2016).

We regressed deaths from each of the 10 cause groups on the drug-coded mortality rate and the lung cancer death rate using the model specification shown in Eq. 2. Drug coefficients (not shown) were mostly negative for other mental/behavioral and ill-defined causes, which may reflect interstate variation in coding (i.e., these are the causes for which we expect there is most likely to be coding misclassification).^[[1]](#footnote-1)^ If coroners in some states are less likely to attribute a death to drug use (instead coding those deaths to other causes), it would create a negative association between drug-coded deaths and deaths from those other causes. We also found a negative association with infectious/parasitic deaths below age 45, although the drug coefficients were strongly positive at ages 50-64. The share of deaths resulting from HIV and viral hepatitis was much higher at younger than at older ages. Thus, the inverse correlation may have resulted from misclassification between drug-related causes and HIV/hepatitis.

Most of the drug coefficients were strongly positive for circulatory, respiratory, and digestive causes, while they were moderately positive for non-drug-related external causes. The drug coefficients were weak, albeit still mostly positive, for non-lung cancer deaths. Surprisingly, the drug coefficients for endocrine/nutritional/metabolic causes were strongly positive among women and moderate among men. We expected the drug coefficients to be weak for the residual category (all other causes), but instead found small to moderate positive associations in women aged 30-49 and men aged 30-64.

When we fit models separately for 10 COD groups, we find that the second largest share of drug-associated deaths—after drug-coded deaths themselves—is circulatory diseases (~20% in 2016; S2 Table). Other causes that account for a substantial share of drug-associated deaths include non-drug-related external causes (10% in men, 5% in women), digestive diseases (7% in men, 10% in women), respiratory diseases (6% in men, 9% in women), and non-lung cancers (8% in men, 4% in women). Although the drug coefficients were weak for non-lung cancers, they contribute a lot of drug-associated deaths because it is a common cause of death. Other mental/behavioral disorders (excluding drug-related) and ill-defined deaths reduce the number of drug-associated deaths (especially among women) because of the negative association with drug-coded mortality.

1. In cases where the drug coefficient was negative, the *reduction* in cause-specific deaths (for a given state-year-sex-age group) as a result of drug use was generally small (five or fewer deaths). The biggest reductions were for circulatory diseases because it is such a common cause of death; the observation with the biggest reduction was females aged 85+ in California in 2007 among whom there were 21,716 circulatory deaths and the estimated drug-associated fraction was -0.013, implying that drug use reduced circulatory deaths by 284. Ill-defined causes were the next biggest source of reductions; the observation with the biggest reduction was men aged 45-49 in California in 2001, among whom there were 521 ill-defined deaths and the estimated drug-associated fraction was -0.065, implying that drug use reduced ill-defined deaths by 34. For all other cause groups, reductions greater than 5 deaths comprised less than 2% of all cases where the drug coefficient was negative. [↑](#footnote-ref-1)
