# Supplementary material for: Estimating the impact of drug use on US mortality, 1999-2016
Source: PLoS One. 2020 Jan 15;15(1):e0226732. doi: 10.1371/journal.pone.0226732 (PMC6961845; doi:10.1371/journal.pone.0226732)
Supplement: S1 Fig — (DOCX) [file pone.0226732.s006.docx]

# S1 Figure. Age-standardized drug-coded mortality rate (per 1,000) for the US, West Virginia, and Nebraska, ages 15 and older, 1981-2016

Note: We plot the trends for the 50 US states as a whole alongside those for the state with the highest (West Virginia) and lowest (Nebraska) drug-coded mortality in 2016. Data for 1981-1998 come from the same sources as the data for 1999-2016: death counts come from the public-use Multiple Cause-of-Death micro-data files (National Center for Health Statistics, 2018) and the corresponding population estimates were downloaded from the CDC Wonder online database (United States Department of Health and Human Services (US DHHS), Centers for Disease Control and Prevention (CDC), National Center for Health Statistics (NCHS), 2018a; United States Department of Health and Human Services (US DHHS), Centers for Disease Control and Prevention (CDC), National Center for Health Statistics (NCHS), 2018b). For the period 1981-1998, we used the following ICD-9 codes to define drug-coded mortality: 304, E850-E858, E950.0-E950.5, E962.0, and E980.0-E980.

Do-File: ~\Google Drive\Professional\Papers\DrugImpact\Do-Files\Describe.do

# References

National Center for Health Statistics. (2018). *Mortality multiple cause-of-death data files.* Retrieved May 3, 2018, from <https://www.cdc.gov/nchs/nvss/mortality_public_use_data.htm>

United States Department of Health and Human Services (US DHHS), Centers for Disease Control and Prevention (CDC), National Center for Health Statistics (NCHS). (2018a). *Bridged-race population estimates, united states july 1st resident population by state, county, age, sex, bridged-race, and hispanic origin. compiled from 1990-1999 bridged-race intercensal population estimates (released by NCHS on 7/26/2004). available on CDC WONDER online database.* Retrieved May 3, 2018, from <http://wonder.cdc.gov/bridged-race-v2016.html>

United States Department of Health and Human Services (US DHHS), Centers for Disease Control and Prevention (CDC), National Center for Health Statistics (NCHS). (2018b). *Intercensal population estimates, NCHS 1981-1989.* Retrieved May 3, 2018, from <https://wonder.cdc.gov/wonder/sci_data/census/inter/type_txt/nchsinte.asp>
