# Supplementary material for: Estimating the impact of drug use on US mortality, 1999-2016
Source: PLoS One. 2020 Jan 15;15(1):e0226732. doi: 10.1371/journal.pone.0226732 (PMC6961845; doi:10.1371/journal.pone.0226732)
Supplement: S1 Table — (DOCX) [file pone.0226732.s007.docx]

# S1 Table. Estimated coefficients from negative binomial regression using drug-coded mortality rate as a predictor, 1999-2016, modeled separately by sex

|  | **Men** | | | | **Women** | | | |
| --- | --- | --- | --- | --- | --- | --- | --- | --- |
|  | Model 1^a^ | | Model 2^b^ | | Model 1^a^ | | Model 2^b^ | |
| **Variable** | β | 95% CI | β | 95% CI | β | 95% CI | β | 95% CI |
| Ages 15-19 | -- |  | -- |  | -- |  | -- |  |
| Ages 20-24 | 0.429 | (0.410, 0.448) | 0.430 | (0.411, 0.448) | 0.230 | (0.208, 0.252) | 0.228 | (0.206, 0.250) |
| Ages 25-29 | 0.394 | (0.370, 0.417) | 0.392 | (0.368, 0.415) | 0.337 | (0.310, 0.364) | 0.333 | (0.305, 0.360) |
| Ages 30-34 | 0.470 | (0.448, 0.493) | 0.464 | (0.442, 0.487) | 0.606 | (0.580, 0.632) | 0.596 | (0.570, 0.623) |
| Ages 35-39 | 0.738 | (0.716, 0.760) | 0.716 | (0.694, 0.738) | 1.008 | (0.982, 1.034) | 0.974 | (0.948, 1.001) |
| Ages 40-44 | 1.130 | (1.108, 1.152) | 1.068 | (1.045, 1.091) | 1.459 | (1.434, 1.484) | 1.385 | (1.359, 1.412) |
| Ages 45-49 | 1.568 | (1.547, 1.590) | 1.429 | (1.403, 1.456) | 1.892 | (1.870, 1.915) | 1.769 | (1.742, 1.797) |
| Ages 50-54 | 1.977 | (1.959, 1.995) | 1.820 | (1.794, 1.846) | 2.317 | (2.297, 2.338) | 2.129 | (2.099, 2.159) |
| Ages 55-59 | 2.401 | (2.385, 2.417) | 2.200 | (2.173, 2.227) | 2.779 | (2.760, 2.798) | 2.536 | (2.504, 2.567) |
| Ages 60-64 | 2.846 | (2.832, 2.861) | 2.666 | (2.640, 2.692) | 3.267 | (3.249, 3.286) | 3.027 | (2.997, 3.058) |
| Ages 65-69 | 3.284 | (3.269, 3.298) | 3.141 | (3.117, 3.165) | 3.708 | (3.691, 3.726) | 3.469 | (3.442, 3.495) |
| Ages 70-74 | 3.720 | (3.705, 3.734) | 3.565 | (3.537, 3.592) | 4.174 | (4.156, 4.191) | 3.949 | (3.918, 3.980) |
| Ages 75-79 | 4.181 | (4.166, 4.195) | 4.105 | (4.073, 4.136) | 4.662 | (4.645, 4.680) | 4.491 | (4.458, 4.525) |
| Ages 80-84 | 4.673 | (4.658, 4.689) | 4.797 | (4.764, 4.829) | 5.191 | (5.173, 5.209) | 5.094 | (5.061, 5.127) |
| Ages 85+ | 5.392 | (5.376, 5.408) | 5.542 | (5.511, 5.573) | 6.087 | (6.069, 6.106) | 6.032 | (6.003, 6.062) |
| AL | -- |  | -- |  | -- |  | -- |  |
| AK | -0.308 | (-0.334, -0.282) | -0.286 | (-0.312, -0.260) | -0.284 | (-0.311, -0.257) | -0.299 | (-0.327, -0.270) |
| AZ | -0.344 | (-0.366, -0.322) | -0.326 | (-0.349, -0.304) | -0.368 | (-0.389, -0.347) | -0.359 | (-0.380, -0.338) |
| AR | -0.055 | (-0.078, -0.032) | -0.062 | (-0.084, -0.041) | -0.061 | (-0.087, -0.035) | -0.082 | (-0.107, -0.056) |
| CA | -0.480 | (-0.498, -0.462) | -0.458 | (-0.475, -0.440) | -0.476 | (-0.497, -0.455) | -0.461 | (-0.482, -0.440) |
| CO | -0.468 | (-0.488, -0.449) | -0.440 | (-0.459, -0.422) | -0.436 | (-0.459, -0.413) | -0.415 | (-0.437, -0.393) |
| CT | -0.523 | (-0.544, -0.502) | -0.509 | (-0.530, -0.489) | -0.484 | (-0.507, -0.462) | -0.491 | (-0.513, -0.469) |
| DE | -0.310 | (-0.330, -0.290) | -0.309 | (-0.328, -0.289) | -0.282 | (-0.304, -0.259) | -0.312 | (-0.334, -0.289) |
| FL | -0.328 | (-0.349, -0.307) | -0.329 | (-0.350, -0.308) | -0.323 | (-0.348, -0.299) | -0.335 | (-0.359, -0.312) |
| GA | -0.178 | (-0.197, -0.160) | -0.171 | (-0.188, -0.154) | -0.144 | (-0.164, -0.123) | -0.138 | (-0.158, -0.118) |
| HI | -0.520 | (-0.539, -0.501) | -0.508 | (-0.529, -0.488) | -0.579 | (-0.601, -0.556) | -0.546 | (-0.570, -0.522) |
| ID | -0.407 | (-0.427, -0.387) | -0.381 | (-0.400, -0.361) | -0.360 | (-0.383, -0.337) | -0.346 | (-0.368, -0.324) |
| IL | -0.339 | (-0.357, -0.322) | -0.331 | (-0.348, -0.314) | -0.322 | (-0.342, -0.301) | -0.330 | (-0.350, -0.311) |
| IN | -0.239 | (-0.257, -0.222) | -0.241 | (-0.257, -0.224) | -0.214 | (-0.234, -0.193) | -0.238 | (-0.257, -0.218) |
| IA | -0.405 | (-0.425, -0.385) | -0.399 | (-0.418, -0.379) | -0.381 | (-0.401, -0.360) | -0.388 | (-0.409, -0.368) |
| KS | -0.320 | (-0.338, -0.302) | -0.310 | (-0.328, -0.293) | -0.290 | (-0.311, -0.270) | -0.297 | (-0.317, -0.277) |
| KY | -0.131 | (-0.150, -0.113) | -0.150 | (-0.167, -0.133) | -0.103 | (-0.124, -0.082) | -0.157 | (-0.177, -0.136) |
| LA | -0.027 | (-0.049, -0.005) | -0.029 | (-0.050, -0.008) | -0.030 | (-0.054, -0.006) | -0.039 | (-0.062, -0.015) |
| ME | -0.368 | (-0.388, -0.348) | -0.365 | (-0.386, -0.345) | -0.343 | (-0.367, -0.319) | -0.378 | (-0.402, -0.353) |
| MD | -0.325 | (-0.345, -0.306) | -0.317 | (-0.336, -0.298) | -0.303 | (-0.324, -0.283) | -0.309 | (-0.328, -0.289) |
| MA | -0.535 | (-0.560, -0.510) | -0.527 | (-0.552, -0.502) | -0.492 | (-0.518, -0.467) | -0.514 | (-0.538, -0.489) |
| MI | -0.312 | (-0.329, -0.295) | -0.308 | (-0.325, -0.292) | -0.271 | (-0.291, -0.251) | -0.289 | (-0.308, -0.270) |
| MN | -0.534 | (-0.555, -0.513) | -0.520 | (-0.541, -0.500) | -0.504 | (-0.527, -0.482) | -0.510 | (-0.533, -0.488) |
| MS | 0.057 | (0.032, 0.081) | 0.051 | (0.028, 0.074) | 0.045 | (0.016, 0.074) | 0.040 | (0.011, 0.068) |
| MO | -0.209 | (-0.227, -0.191) | -0.210 | (-0.228, -0.193) | -0.199 | (-0.219, -0.179) | -0.224 | (-0.244, -0.205) |
| MT | -0.315 | (-0.338, -0.292) | -0.290 | (-0.314, -0.267) | -0.304 | (-0.328, -0.281) | -0.312 | (-0.335, -0.289) |
| NE | -0.387 | (-0.406, -0.368) | -0.374 | (-0.392, -0.355) | -0.361 | (-0.383, -0.340) | -0.358 | (-0.379, -0.337) |
| NV | -0.280 | (-0.298, -0.261) | -0.259 | (-0.277, -0.240) | -0.253 | (-0.274, -0.232) | -0.277 | (-0.298, -0.256) |
| NH | -0.481 | (-0.505, -0.457) | -0.474 | (-0.498, -0.450) | -0.424 | (-0.450, -0.398) | -0.454 | (-0.481, -0.427) |
| NJ | -0.448 | (-0.468, -0.428) | -0.431 | (-0.450, -0.411) | -0.396 | (-0.418, -0.375) | -0.397 | (-0.417, -0.376) |
| NM | -0.266 | (-0.295, -0.237) | -0.238 | (-0.268, -0.208) | -0.318 | (-0.342, -0.294) | -0.285 | (-0.308, -0.261) |
| NY | -0.469 | (-0.489, -0.449) | -0.457 | (-0.477, -0.437) | -0.430 | (-0.452, -0.408) | -0.430 | (-0.452, -0.408) |
| NC | -0.228 | (-0.245, -0.211) | -0.227 | (-0.243, -0.211) | -0.218 | (-0.238, -0.198) | -0.224 | (-0.243, -0.204) |
| ND | -0.375 | (-0.396, -0.354) | -0.354 | (-0.375, -0.333) | -0.414 | (-0.438, -0.390) | -0.403 | (-0.427, -0.379) |
| OH | -0.292 | (-0.311, -0.274) | -0.294 | (-0.311, -0.276) | -0.239 | (-0.259, -0.218) | -0.257 | (-0.277, -0.237) |
| OK | -0.113 | (-0.132, -0.093) | -0.111 | (-0.129, -0.092) | -0.068 | (-0.090, -0.046) | -0.088 | (-0.109, -0.066) |
| OR | -0.418 | (-0.437, -0.400) | -0.402 | (-0.421, -0.383) | -0.365 | (-0.387, -0.343) | -0.380 | (-0.401, -0.359) |
| PA | -0.333 | (-0.350, -0.315) | -0.325 | (-0.342, -0.308) | -0.318 | (-0.338, -0.298) | -0.325 | (-0.345, -0.306) |
| RI | -0.444 | (-0.467, -0.421) | -0.440 | (-0.463, -0.416) | -0.413 | (-0.436, -0.389) | -0.442 | (-0.466, -0.418) |
| SC | -0.096 | (-0.118, -0.075) | -0.097 | (-0.117, -0.077) | -0.119 | (-0.143, -0.096) | -0.121 | (-0.144, -0.098) |
| SD | -0.333 | (-0.354, -0.312) | -0.313 | (-0.334, -0.292) | -0.377 | (-0.401, -0.354) | -0.374 | (-0.397, -0.351) |
| TN | -0.101 | (-0.120, -0.082) | -0.110 | (-0.128, -0.093) | -0.097 | (-0.118, -0.076) | -0.119 | (-0.139, -0.098) |
| TX | -0.286 | (-0.303, -0.269) | -0.268 | (-0.285, -0.251) | -0.256 | (-0.276, -0.237) | -0.243 | (-0.262, -0.223) |
| UT | -0.519 | (-0.541, -0.498) | -0.490 | (-0.511, -0.470) | -0.430 | (-0.457, -0.404) | -0.365 | (-0.391, -0.339) |
| VT | -0.447 | (-0.472, -0.423) | -0.444 | (-0.468, -0.419) | -0.390 | (-0.416, -0.365) | -0.412 | (-0.439, -0.385) |
| VA | -0.361 | (-0.379, -0.343) | -0.351 | (-0.369, -0.333) | -0.315 | (-0.335, -0.295) | -0.317 | (-0.336, -0.297) |
| WA | -0.491 | (-0.512, -0.471) | -0.478 | (-0.498, -0.458) | -0.438 | (-0.461, -0.414) | -0.452 | (-0.474, -0.430) |
| WV | -0.130 | (-0.149, -0.111) | -0.137 | (-0.155, -0.119) | -0.091 | (-0.113, -0.069) | -0.125 | (-0.146, -0.103) |
| WI | -0.428 | (-0.448, -0.409) | -0.415 | (-0.435, -0.396) | -0.408 | (-0.429, -0.386) | -0.415 | (-0.436, -0.394) |
| WY | -0.313 | (-0.336, -0.290) | -0.281 | (-0.305, -0.257) | -0.272 | (-0.295, -0.250) | -0.261 | (-0.284, -0.239) |
| Year - 1999 | -0.015 | (-0.016, -0.015) | -0.014 | (-0.015, -0.014) | -0.013 | (-0.013, -0.013) | -0.012 | (-0.013, -0.012) |
| Drug-coded death rate $(M_{D})$ | 0.184 | (0.081, 0.287) | 0.174 | (0.071, 0.277) | 0.235 | (-0.030, 0.501) | 0.263 | (-0.002, 0.528) |
| $M_{D}\times(\text{Age} 25-29)$ | 0.133 | (0.010, 0.256) | 0.140 | (0.018, 0.262) | 0.507 | (0.196, 0.819) | 0.499 | (0.188, 0.810) |
| $M_{D}\times(\text{Age} 30-34)$ | 0.167 | (0.047, 0.287) | 0.174 | (0.054, 0.294) | 0.536 | (0.243, 0.829) | 0.523 | (0.230, 0.816) |
| $M_{D}\times(\text{Age} 35-39)$ | 0.105 | (-0.011, 0.221) | 0.124 | (0.008, 0.240) | 0.350 | (0.068, 0.633) | 0.379 | (0.097, 0.662) |
| $M_{D}\times(\text{Age} 40-44)$ | 0.055 | (-0.060, 0.169) | 0.089 | (-0.027, 0.204) | 0.211 | (-0.065, 0.488) | 0.251 | (-0.026, 0.528) |
| $M_{D}\times(\text{Age} 45-49)$ | 0.069 | (-0.044, 0.183) | 0.136 | (0.022, 0.249) | 0.236 | (-0.034, 0.506) | 0.234 | (-0.036, 0.505) |
| $M_{D}\times(\text{Age} 50-54)$ | 0.205 | (0.095, 0.315) | 0.285 | (0.175, 0.395) | 0.279 | (0.009, 0.549) | 0.264 | (-0.005, 0.533) |
| $M_{D}\times(\text{Age} 55-59)$ | 0.245 | (0.134, 0.356) | 0.398 | (0.284, 0.512) | 0.098 | (-0.174, 0.371) | 0.204 | (-0.067, 0.475) |
| $M_{D}\times(\text{Age} 60-64)$ | 0.136 | (0.020, 0.253) | 0.351 | (0.230, 0.471) | -0.312 | (-0.589, -0.034) | -0.049 | (-0.330, 0.232) |
| $M_{D}\times(\text{Age} 65+)$ | -0.287 | (-0.404, -0.170) | -0.250 | (-0.368, -0.133) | -0.368 | (-0.641, -0.096) | -0.379 | (-0.651, -0.107) |
| Lung cancer death rate $(M_{L})$ | -- |  | 0.348 | (0.282, 0.414) | -- |  | 0.306 | (0.202, 0.410) |
| $M_{L}\times(\text{Age} 50-54)$ | -- |  | -0.210 | (-0.277, -0.143) | -- |  | -0.012 | (-0.131, 0.106) |
| $M_{L}\times(\text{Age} 55-59)$ | -- |  | -0.269 | (-0.333, -0.204) | -- |  | -0.111 | (-0.217, -0.005) |
| $M_{L}\times(\text{Age} 60-64)$ | -- |  | -0.324 | (-0.389, -0.260) | -- |  | -0.214 | (-0.317, -0.111) |
| $M_{L}\times(\text{Age} 65-69)$ | -- |  | -0.345 | (-0.409, -0.280) | -- |  | -0.236 | (-0.339, -0.134) |
| $M_{L}\times(\text{Age} 70-74)$ | -- |  | -0.340 | (-0.405, -0.274) | -- |  | -0.256 | (-0.359, -0.153) |
| $M_{L}\times(\text{Age} 75-79)$ | -- |  | -0.353 | (-0.419, -0.288) | -- |  | -0.273 | (-0.377, -0.170) |
| $M_{L}\times(\text{Age} 80+)$ | -- |  | -0.385 | (-0.451, -0.319) | -- |  | -0.289 | (-0.394, -0.185) |
| Constant | -6.746 | (-6.767, -6.726) | -6.763 | (-6.783, -6.743) | -7.642 | (-7.667, -7.618) | -7.645 | (-7.670, -7.621) |
| N | 13,500 |  | 13,500 |  | 13,500 |  | 13,500 |  |

Note: Standard errors are computed using the robust (Huber/White/sandwich) variance estimator.

^a^ Model 1 regresses the death rate from causes other than drugs on the drug-coded mortality rate $(M_{D})$; see Eq. 1 in S1 Appendix. The main effect of $M_{D}$ represents the drug coefficient for persons aged 15-24. The drug coefficients for other age groups (as shown in Table 1) can be obtained by summing the main effect and the interaction between $M_{D}$and the corresponding age group. For example, the drug coefficent for men aged 55-59 (0.429) is the sum of the main effect (0.184) and the interaction effect (0.245).

^b^ Model 2 adds lung cancer mortality as a predictor and the outcome is modified to represent the mortality rate from causes other than drugs or lung cancer (see Eq. 2 in S2 Appendix).

Do-File: ~\Google Drive\Professional\Papers\DrugImpact\Do-Files\Model.do
