# Supplementary material for: Estimating the impact of drug use on US mortality, 1999-2016
Source: PLoS One. 2020 Jan 15;15(1):e0226732. doi: 10.1371/journal.pone.0226732 (PMC6961845; doi:10.1371/journal.pone.0226732)
Supplement: S2 Table — (DOCX) [file pone.0226732.s008.docx]

# S2 Table. Estimated number of drug-associated deaths among those aged 15-64 and distribution across groups of causes by sex, US, 2016

|  | **Drug-Associated**  **Deaths, No.** | | **% Distribution**  **by Cause Group** | |
| --- | --- | --- | --- | --- |
|  | Men | Women | Men | Women |
| Based on Model 2 (all other causes combined) | 98,508 | 43,187 |  |  |
| Based on cause-specific models | 100,413 | 46,436 |  |  |
| **By cause group**^a^ |  |  |  |  |
| Drug-coded deaths | 41,501 | 21,499 | 41.3 | 46.3 |
| Mental & behavioral disorders (excluding drug-related) | -505 | -446 | -0.5 | -1.0 |
| Ill-defined causes | -267 | -704 | -0.3 | -1.5 |
| Infectious & parasitic diseases | 2,934 | 942 | 2.9 | 2.0 |
| Respiratory diseases | 5,724 | 4,162 | 5.7 | 9.0 |
| Digestive diseases | 6,705 | 4,517 | 6.7 | 9.7 |
| External causes (excluding drug-related) | 10,117 | 2,404 | 10.1 | 5.2 |
| Circulatory diseases | 20,068 | 9,600 | 20.0 | 20.7 |
| Neoplasms (excluding lung cancer) | 8,254 | 2,013 | 8.2 | 4.3 |
| Endocrine, nutritional, & metabolic diseases | 3,510 | 2,403 | 3.5 | 5.2 |
| All other causes | 2,371 | 45 | 2.4 | 0.1 |

^a^ See S5 Appendix for ICD-10 codes that define each cause group. Negative values result when the model indicates an inverse association between drug-coded mortality and mortality from the specified cause group.
