# Supplementary material for: Estimating the impact of drug use on US mortality, 1999-2016
Source: PLoS One. 2020 Jan 15;15(1):e0226732. doi: 10.1371/journal.pone.0226732 (PMC6961845; doi:10.1371/journal.pone.0226732)
Supplement: S3 Table — (DOCX) [file pone.0226732.s009.docx]

# S3 Table. Estimated difference in life expectancy at age 15 (*e*_15_) and percentage dying between age 15 and 65 (*q*[15,65]) associated with drug use, by state and sex, 2016

|  | **Difference in** *e*_15_ **(years)^a^** | |  | **Difference in *q*[15,65]** **(%)^b^** | |
| --- | --- | --- | --- | --- | --- |
| State | Men | Women |  | Men | Women |
| Alabama | 1.2 | 0.7 |  | 3.2 | 1.9 |
| Alaska | 1.2 | 0.8 |  | 3.2 | 2.0 |
| Arizona | 1.7 | 0.7 |  | 4.6 | 1.9 |
| Arkansas | 1.0 | 0.7 |  | 2.8 | 2.0 |
| California | 0.9 | 0.4 |  | 2.8 | 1.1 |
| Colorado | 1.1 | 0.6 |  | 3.0 | 1.6 |
| Connecticut | 1.9 | 0.7 |  | 5.1 | 1.7 |
| Delaware | 2.2 | 1.0 |  | 5.6 | 2.3 |
| Florida | 1.8 | 0.9 |  | 4.6 | 2.1 |
| Georgia | 1.0 | 0.5 |  | 2.9 | 1.4 |
| Hawaii | 1.2 | 0.4 |  | 3.7 | 1.1 |
| Idaho | 0.9 | 0.6 |  | 2.5 | 1.7 |
| Illinois | 1.4 | 0.5 |  | 3.8 | 1.4 |
| Indiana | 1.6 | 0.9 |  | 4.2 | 2.3 |
| Iowa | 0.7 | 0.3 |  | 1.9 | 1.0 |
| Kansas | 0.7 | 0.5 |  | 2.1 | 1.3 |
| Kentucky | 2.3 | 1.4 |  | 6.6 | 3.7 |
| Louisiana | 1.6 | 0.9 |  | 4.6 | 2.4 |
| Maine | 1.9 | 0.9 |  | 4.7 | 2.1 |
| Maryland | 2.5 | 0.9 |  | 6.9 | 2.3 |
| Massachusetts | 2.3 | 0.8 |  | 6.0 | 2.0 |
| Michigan | 1.8 | 1.0 |  | 5.0 | 2.4 |
| Minnesota | 0.8 | 0.4 |  | 2.1 | 0.9 |
| Mississippi | 1.0 | 0.6 |  | 2.9 | 1.7 |
| Missouri | 1.6 | 0.9 |  | 4.3 | 2.2 |
| Montana | 0.7 | 0.7 |  | 1.8 | 1.9 |
| Nebraska | 0.4 | 0.3 |  | 1.1 | 0.9 |
| Nevada | 1.5 | 0.9 |  | 4.5 | 2.5 |
| New Hampshire | 2.5 | 1.0 |  | 5.8 | 2.3 |
| New Jersey | 1.6 | 0.6 |  | 4.1 | 1.5 |
| New Mexico | 2.0 | 1.1 |  | 5.1 | 2.8 |
| New York | 1.4 | 0.5 |  | 3.7 | 1.2 |
| North Carolina | 1.3 | 0.8 |  | 3.4 | 2.0 |
| North Dakota | 0.7 | 0.4 |  | 1.8 | 1.1 |
| Ohio | 2.6 | 1.3 |  | 7.0 | 3.2 |
| Oklahoma | 1.5 | 1.0 |  | 4.7 | 2.8 |
| Oregon | 0.9 | 0.4 |  | 2.7 | 1.3 |
| Pennsylvania | 2.5 | 1.2 |  | 6.5 | 2.8 |
| Rhode Island | 2.0 | 0.9 |  | 5.5 | 2.2 |
| South Carolina | 1.4 | 0.7 |  | 3.9 | 2.0 |
| South Dakota | 0.5 | 0.4 |  | 1.3 | 1.0 |
| Tennessee | 1.7 | 1.2 |  | 5.0 | 3.1 |
| Texas | 0.7 | 0.4 |  | 2.3 | 1.1 |
| Utah | 1.4 | 0.9 |  | 4.0 | 2.4 |
| Vermont | 1.3 | 0.7 |  | 2.8 | 1.7 |
| Virginia | 1.1 | 0.5 |  | 3.0 | 1.4 |
| Washington | 1.0 | 0.5 |  | 3.0 | 1.5 |
| West Virginia | 3.6 | 1.9 |  | 9.8 | 4.6 |
| Wisconsin | 1.2 | 0.7 |  | 3.0 | 1.7 |
| Wyoming | 0.8 | 0.9 |  | 2.2 | 2.3 |
| All 50 States | 1.4 | 0.7 |  | 3.9 | 1.8 |

^a^ Represents the loss in life expectancy at age 15 ($e_{15}$) associated with drug use, computed as the estimated $e_{15}^{-D}$ in the absence of drug use minus the observed $e_{15}$_._

^b^ Represents the probability of dying between age 15 and 65 $(q\left[ 15,65 \right])$ as a result of drug use (expressed as a percentage), computed as the estimated $q^{-D}\left[ 15,65 \right]$ in the absence of drug use minus the observed $q\left[ 15,65 \right]$ multiplied by 100.

Do-File: ~\Google Drive\Professional\Papers\DrugImpact\Do-Files\LifeTable.do
