# Supplementary material for: Estimating the impact of drug use on US mortality, 1999-2016
Source: PLoS One. 2020 Jan 15;15(1):e0226732. doi: 10.1371/journal.pone.0226732 (PMC6961845; doi:10.1371/journal.pone.0226732)
Supplement: S4 Table — (DOCX) [file pone.0226732.s010.docx]

# S4 Table. Interstate correlations between changes (2016-1999) in drug-coded mortality and corresponding changes in alcohol-related mortality and non-drug suicide rates by age and sex

|  | **Correlation between drug-coded mortality and:** | | | | |
| --- | --- | --- | --- | --- | --- |
|  | Alcohol-related mortality^a^ | |  | Non-Drug suicide rates^b^ | |
| **Age group** | Men | Women |  | Men | Women |
| 15-19 | -0.17 | 0.02 |  | -0.18 | -0.26 |
| 20-24 | -0.19 | 0.09 |  | 0.05 | -0.47 |
| 25-29 | -0.16 | -0.22 |  | -0.28 | 0.07 |
| 30-34 | -0.08 | 0.07 |  | 0.16 | 0.18 |
| 35-39 | 0.01 | 0.29 |  | 0.02 | 0.23 |
| 40-44 | 0.10 | 0.10 |  | 0.04 | -0.06 |
| 45-49 | 0.05 | 0.08 |  | -0.09 | 0.30 |
| 50-54 | -0.15 | -0.28 |  | -0.07 | 0.06 |
| 55-59 | -0.04 | -0.06 |  | 0.34 | -0.24 |
| 60-64 | -0.10 | 0.13 |  | -0.38 | -0.14 |
| 65-69 | -0.18 | -0.19 |  | 0.12 | -0.01 |
| 70-74 | 0.14 | 0.01 |  | -0.10 | -0.20 |
| 75-79 | 0.04 | -0.11 |  | 0.13 | 0.35 |
| 80-84 | -0.02 | -0.43 |  | -0.10 | -0.21 |
| 85+ | -0.35 | -0.03 |  | 0.11 | 0.04 |

^a^ Alcohol-related mortality includes ICD-10 codes alcohol poisonings (X45, Y15), chronic liver disease and cirrhosis (K70, K73-K74), and alcohol-related mental/behavioral disorders.

^b^ Non-drug suicides include ICD-10 codes X65-X84 and Y87.0.

Note: Negative correlations are shaded in grey.

Do-File: ~\Google Drive\Professional\Papers\DrugImpact\Do-Files\Describe.do
